# A Multi-Level Output-Based DBN Model for Fine Classification of Complex Geo-Environments Area Using Ziyuan-3 TMS Imagery

**DOI:** 10.3390/s21062089

**Published:** 2021-03-16

**Authors:** Meng Li, Zhuang Tang, Wei Tong, Xianju Li, Weitao Chen, Lizhe Wang

**Affiliations:** 1 Faculty of Computer Science, China University of Geosciences, Wuhan 430074, China; mengli@cug.edu.cn (M.L.); tangzhuang@cug.edu.cn (Z.T.); weitong@cug.edu.cn (W.T.); ddwhlxj@cug.edu.cn (X.L.); lzwang@cug.edu.cn (L.W.); 2 Hubei Key Laboratory of Intelligent Geo-Information Processing, China University of Geosciences, Wuhan 430074, China

**Keywords:** remote sensing, deep learning, fine-scale classification, deep belief networks, open-pit mining, Ziyuan-3 imagery

## Abstract

Fine-scale land use and land cover (LULC) data in a mining area are helpful for the smart supervision of mining activities. However, the complex landscape of open-pit mining areas severely restricts the classification accuracy. Although deep learning (DL) algorithms have the ability to extract informative features, they require large amounts of sample data. As a result, the design of more interpretable DL models with lower sample demand is highly important. In this study, a novel multi-level output-based deep belief network (DBN-ML) model was developed based on Ziyuan-3 imagery, which was applied for fine classification in an open-pit mine area of Wuhan City. First, the last DBN layer was used to output fine-scale land cover types. Then, one of the front DBN layers outputted the first-level land cover types. The coarse classification was easier and fewer DBN layers were sufficient. Finally, these two losses were weighted to optimize the DBN-ML model. As the first-level class provided a larger amount of additional sample data with no extra cost, the multi-level output strategy enhanced the robustness of the DBN-ML model. The proposed model produces an overall accuracy of 95.10% and an F1-score of 95.07%, outperforming some other models.

## 1. Introduction

It is well known that land use and land cover mapping and its consequences for eco-environmental impact on Earth has been increasingly critical for sustainable development. Open-pit mining is a high-intensity human activity, which significantly impacts a mining area and its surrounding environment [1,2,3]. The mining area and surrounding cropland, forestland, and other surface environmental elements are considered to constitute a complex geological environment. In these areas, a series of geological environmental problems may occur [4,5]. For example, they can cause land degradation [1,2,3,4,5], groundwater pollution, decreased vegetation cover, soil pollution, and geological disasters [6,7,8]. In general, the land use and land cover (LULC) related to open-pit mining is the key in these complex environment areas. In open-pit mining areas, the land use and land cover (LULC) classification represents an important basis for environmental assessment and protection, as well as ground deformation monitoring [6,9,10,11]. Owing to issues and challenges related to LULC [12,13] and the wide application of LULC data as the basic input in interdisciplinary studies [14], LULC has been a popular research target in high-resolution remote sensing techniques [14,15]. However, the complexity and diversity of terrain characteristics in open-pit mining areas, such as enhanced three-dimensional (3-D) terrain features [16,17] and intense spatiotemporal variability [5,18] reduce the fine LULC (FLULC) accuracy, limiting the application of remote sensing technology in the geo-environmental monitoring of mining areas. 

At present, multi-source remote sensing data fusion is among the most studied methods of image interpretation in remote sensing [19]. Comparisons of mining area classification results based on spectral information with integrated digital elevation model (DEM) and spectral information data reveal superior results from the latter [20]. Machine-learning algorithms (MLAs) accommodate varied feature sets, with algorithms, such as the support vector machine (SVM) [21,22,23,24] and random forest (RF) [22], widely employed for LULC classification in complex mining areas [18,25,26,27,28]. Chen et al. [29] highlighted the importance of obtaining remote sensing features and developing an effective classification model for fine LULC classification. Li et al. [26] carried out fine LULC classification in open-pit mining areas, revealing that MLAs can provide improved classification performance. Qu et al. [30] improved the classification accuracy by extracting the soil characteristics and phenological characteristics of the auxiliary dataset from the Google Earth Engine. Yao et al. [31] studied the advantages of using continuous multi-angle remote sensing data for classification, attempting to make use of the complementarity of multi-angle information. Zhang et al. [32] proposed a weak to strong supervised learning framework for LULC classification to solve the absence of well-labeled and abundant pixel-level annotations. Tan et al. [33] considered the appropriate application of large-scale spectral clustering to conform to substantial land and complex terrain characteristics. Li et al. [34] predicted the LULC using the integrated logistic-cellular automata-Markov chain model. Su et al. [35] combined pixel swapping and simulated annealing to obtain spatial information at the subpixel level for land cover mapping. Zhao et al. [36] used the relative utility of each spectral band and the spectrally weighted kernel to improve the classification performance. MLAs perform well in remote sensing classification by combining various effective features.

Furthermore, deep learning algorithms are increasingly popular in remote sensing classification because depth and discriminating features can be extracted layer-by-layer [37,38]. Zhang et al. [39] proposed a scale sequence joint deep learning method by incorporating a sequence of scales in a single unified modeling framework for LULC classification. Chen et al. [37] proposed a novel attention-driven context encoding network method for coastal land cover classification from high-resolution remote sensing images. The deep belief network (DBN), involving unsupervised learning in feature extraction, is among the most commonly utilized algorithms, with great successes achieved in image recognition, information retrieval, and natural language processing, among others [40,41]. However, the demand for labeled data remains low. Nevertheless, DBN and DBN-based frameworks have been used for selecting remote sensing scene classification features [42]. Zhao et al. [43], for example, constructed an unsupervised feature learning method to classify synthetic aperture radar imagery using a DBN and an ensemble learning algorithm. Meanwhile, Ayhan and Kwan [44] compared the use of a DBN algorithm with those of the spectral angle mapper and SVM. In addition, Chen et al. [45] proposed spatial, spectral, and spectral–spatial feature-based DBN paradigms for hyperspectral imagery classification, integrating the DBN and logistic regression (LR) to produce a DBN-LR algorithm [45,46]. Chen et al. [47] applied a DBN model for remote sensing image classification. Furthermore, Zhong et al. [48] proposed a model by integrating the DBN and conditional random field for hyperspectral image classification. Moreover, Qin et al. [49] constructed a model combining the restricted Boltzmann machine (RBM) and an adaptive boosting (AdaBoost) method. He et al. [50] also integrated a deep stacking network (DSN), which is similar to the DBN, with the LR to create a DSN-LR algorithm. The DBN has been widely applied in the remote sensing field. However, few studies have focused on the fine classification of the complex LULC in open-pit mining areas based on the DBN. Although the DBN-based multimodal and multi-model deep fusion method using training, validation, and test sets based on spatial autocorrelation have yielded remarkable performance [51], further strategies for improving fine classification are required.

The main objective of this study was to construct a multi-level output-based deep belief network (DBN-ML) model for the fine classification of complex environments using Ziyuan-3 (ZY-3) TMS data. The contributions of this study are as follows: (1) the last DBN layer was used to output fine-scale land cover types; (2) one of the front DBN layers outputted the first-level land cover types. The coarse classification was easier and fewer DBN layers were sufficient. (3) These two losses were weighted to optimize the DBN-ML model. As the first-level class provided a larger amount of additional sample data with no extra cost, the multi-level output strategy enhanced the robustness of the DBN-ML model. This study can aid in the effective supervision of mining activities at local or regional scales.

## 2. Methods

### 2.1. Study Area and Remote Sensing Data

The study area, covering an area of 109.4 km^2^, is in the Jiangxia district of Wuhan City in Hubei province, China. This area exhibits the typical characteristics of a mining and agricultural area, with a large open-pit mining area known as the Wulongquan mining area. Currently, some mines in the study area are non-operational, but several mines remain operational. The activities in the operational areas mainly involve mining, beneficiation, and ore-washing.

**Figure 1:**
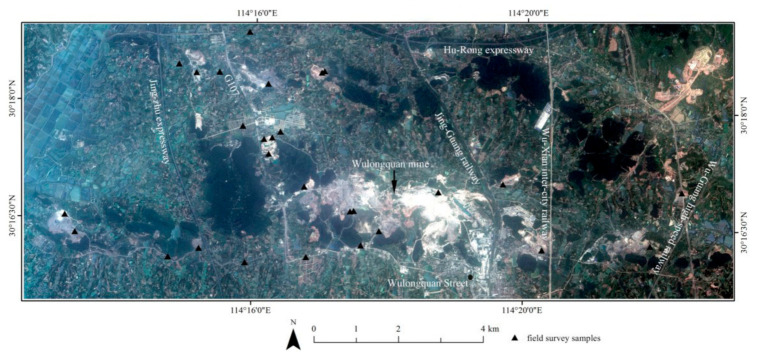
Image showing the study area and field sampling locations (revised from [51]).

The ZY-3 is an independent and civilian high-resolution stereo mapping satellite launched by China on January 9, 2012. The satellite captured panchromatic (PAN) and multispectral images. The multispectral imagery resolution was 5.8 m, the nadir PAN image was 2.1 m, and the forward and backward images were 3.5 m. The forward and backward images at 3.5 m were used to generate the DEM data at 10 m. The spectral resolutions are as follows: Blue (450–520 nm), Green (520–590 nm), Red (630–690 nm), NIR (770–890 nm), and PAN (450–800 nm). The ZY-3 imagery used are level 1B products captured on 20 June 2012; Figure 1 displays the image of the study area.

According to the mining environment monitoring requirements in China, the LULC types in the study area were divided into first-level and second-level categories. The seven first-level categories are road, water, arable land, urban and rural residence, construction land, forest land, unused land, and mine objects. The 20 secondary categories obtained from a previous study [26] are presented in Table 1.

### 2.2. DBN-Based Multi-Level Classification Model Construction

The DBN was constructed based on RBM stacking [41], comprising Boltzmann machines and a back propagation network layer. For each limited Boltzmann machine, the training was unsupervised, whereas fine tuning of the full network by the completed DBN was supervised. The DBN enables unsupervised training of each RBM layer and mapping of feature vectors in different spaces, with some achievements in remote sensing classification. When the number of RBM layers and nodes in DBN are smaller, the model fitting ability is insufficient and the classification accuracy are not good. Thus, the DBN requires a certain network depth (more RBM layers and more nodes). However, when the DBN network is deeper, the gradient disappears easily during the training process, which makes it difficult to adjust the parameters of the first few layers of the network, thereby restricting the further improvement of the classification accuracy. 

However, in open-pit mining areas, the LULC types of the ground objects are complex, with significantly different spatial geometric characteristics for the ground objects and discontinuous image patches. These complexities often cause over-fitting, thereby limiting the generalization performance of a DBN model [52]. As many scale features for the ground surface objects exist, multi-scale feature extraction can be used to enhance the classification accuracy. Multi-scale features extraction for complex surface conditions has been demonstrated to produce better results when extracting different sizes of shallow features (texture and filtering, among others) from remote sensing imagery of mining areas.

**Figure 2:**
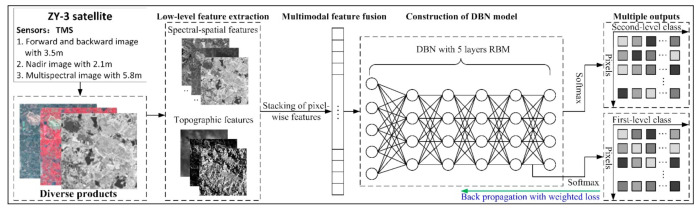
Summary of the multi-level output classification based on the deep belief network (DBN) model proposed in this study.

To solve the issue, we constructed a DBN-ML involving the structure displayed in Figure 2. The main idea of the model is as follows. The DBN-ML may output two-level classification, with the output set at the last layer as a second-level classification (i.e., the second level output) and the output of one layer from the first layer to the penultimate layer as a first-level classification (i.e., the first level output). The classification accuracy of the first-level classification is here less than that of the second-level classification. The first-level classification is a high-level category of ground objects, such as cultivated land, forest, etc., and the second-level classification is the low-level category of ground objects, such as dry land and paddy field in cultivated land. The loss of each of the outputs is calculated based on each of the outputs and the corresponding labels of the outputs, and two outputs will result in two losses. Through provision of two outputs, in which one of the outputs is set in the previous layers of the network (the first layer to the penultimate layer) to introduce the losses of the previous layers into the backpropagation such that the backpropagation may take into account the learning status of the parameters of the previous layers of the network; when the learning of the parameters of the previous layers is rough, the output losses of the previous layers will be larger, and the total loss will also increase after weighting accordingly, so that the phenomenon of gradient disappearance is not easy to occur in the backpropagation, and the network parameters of the previous layers are adjusted accordingly, thereby improving the classification accuracy.

The configuration and all details are as follows. First, low-level features, including the multi-scale topographic and spatial-spectral features, were obtained from the ZY-3 data. Second, multiple features were considered as input for the DBN. The DBN model comprises five layers RBMs and the output results were processed using the SoftMax function. 

The main innovations of this method are as follows: (1) for fine LULC, the first- and second-level land covers are set as the outputs of the DBN-based model; (2) the corresponding losses are weighted for optimizing the model; and (3) only the previous DBN layer is employed to generate the first-level output. 

Besides, single output results based on DBN models and MLAs from our previous study [51] were used for comparison. 

### 2.3. Remote Sensing Features, Training, Validation, and Test Sets

First, multi-source and multi-scale shallow features extracted from products of the ZY-3 imagery were categorized into six types [26]: 1. multiple spectral bands; 2. plant cover; 3. multivariate principal components; 4. multi-scale filtering features; 5. multi-scale texture features; and 6. multiple terrain features, amounting to 106 features. A summary of the low-level features used in this study can be found in literature [26].

Spatially-independent test sets were employed to assess the accuracy [51]. From the 20 second-level feature types in the study area, 2000 sampling points (pixels) were selected for the training set, 500 for the verification set, and 500 for the test set for each category. The fractions representing each set and the associated data polygons are presented in Table 2. The data polygons [51] were constructed based on the following: (1) the polygons in Li et al. [26] and (2) the addition of polygons for the mine pit pond, red roof, dark road, and blue roof classes using the visual interpretation approach. 

### 2.4. Comparison of the Deep Learning Feature Algorithm

To further prove the performance of the DBN-ML algorithm, our previous DBN related studies [51] and a novel deformable convolutional neural network (DCNN) [53] were introduced as a comparison. The traditional convolutional neural network (CNN) itself has a rich feature-expression and learning ability, which achieves good effect on mapping rice paddies in complex landscapes with CNN [54]. However, owing to the fixed geometric transformation ability in its module, it has limitations in adapting to different geometric features. Hence, when the network performs fine classification under complex surface conditions, the performance of the model is limited. In the computer vision field, researchers from the Visual Computer Group of Microsoft Asia Research Institute first introduced the ability to learn spatial geometric deformation in CNNs in 2017 [53], successfully performing semantic segmentation and target recognition. The DCNN have recently been demonstrated to be a powerful tool for hyperspectral image (HSI) classification [28]. Therefore, we not only used the traditional CNN (popular VGG) to perform comparative experiments, but we also added the DCNN, which replaces convolution in the VGG with deformable convolution.

### 2.5. Accuracy Evaluation Criteria

In this study, the overall accuracy (OA), kappa coefficient, and F1-score were used to characterize the model quality. In addition, the precision, recall, and F1-measure of each category were analyzed to evaluate the DBN-ML model.

## 3. Results and Discussion

### 3.1. Parameter Optimization Results

#### 3.1.1. DBN Basic Parameters

The number of RBM networks, nodes in the hidden layer, activation functions, iterations, and dropouts, among others, affect the performance of the DBN. The main parameter optimization results from our previous study [51] were directly employed in this study. This involved the parameter combination of five RBMs with 1500 nodes in each layer while the other parameters were set as follows: sigmoid activation function, learning rate of 0.0001, 800 iterations, and mini batch size of 512. 

#### 3.1.2. Loss Weighting Results

After selecting the basic DBN structure, the weighting scheme of the two output-based losses were determined. Initially, the fourth layer was used to generate the first-level land classes while the last layer produced the second-level classes. The weights of the first-level classes were set as 0.1 to 0.9 while those for the second-level were set from 0.9 to 0.1. Each combination was run five times. The results show an optimal weight combination of 0.2 for the first-level classes and 0.8 for the second-level classes, with an OA of 94.85% ± 0.17%.

### 3.2. Classification Result Analysis and Evaluation

#### 3.2.1. Visual Analysis of the Classification Map of the Entire Study Area

**Figure 3:**
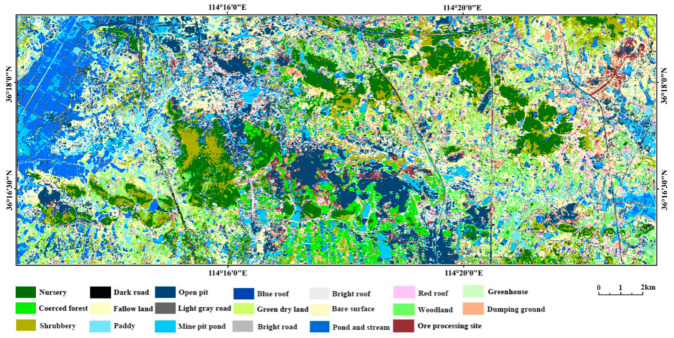
Classification results based on the DBN-ML for the entire study area.

The DBN-ML model was used for predicting the entire study area; Figure 3 shows the prediction results.

Figure 3 reveals an adequate classification of the overall outline of the ground features, with better effects on the forestland, water body, arable land, and mining area. The roads are also well-distinguished in general, although many roads are misclassified as construction land. The two main reasons that accounting for the prediction issues for the entire study area are as follows. 

(1) As the training, verification, and test set samples were randomly selected, these samples are not fully independent, which may cause the samples to be less representative, and

(2) The distributions of the training, verification, and test set samples differ from that of the entire study area. When training the datasets, the multi-source and multi-scale features are normalized, producing an identical number of samples in each category, with such data characterized by a uniform distribution. In contrast, when predicting the entire study area, the number of each category differs, with some major differences. As such data involve a different distribution type, normalization will produce bias.

#### 3.2.2. Classification Accuracy Assessment

Table 3 presents the classification results for each secondary class for a testing dataset using the DBN-ML model based on parameter adjustment, considering the recall, precision, and F1-measure as indicators, as well as the average classification accuracy. The DBN-ML proposed in this study produces notably better results using the test set. 

Based on Table 3, the F1-measure for the DBN-ML models generally exceeds 90%, with only the bright roof showing a value less of than 90%. This low value is due to similar ground features in the spectrum and the spatial structure of these ground features, causing erroneous sample classification, especially for secondary features under the same ground features level. The other 19 features exhibit F1 scores greater than 90%, with the mine water representing the best, reaching 99%. The output results demonstrate that the boundaries of the ground objects can be clearly distinguished.

## 4. Discussion

To reveal the performance of our proposed DBN-ML model, several different models was compared together in this study. The comparison involves four our previous study [51], and they are the FS-SVM, DBN-based models (DBN-S, DBN-SVM, and DBN-RF). In addition, the CNN and DCNN are carried out to compare in this study.

The results of these models were listed in Table 4. In addition, the running time of was DBN was about 31 min while the running time of the DBN-ML was about 65 min. Although the running time of the DBN-ML was usually approximately twice that of the DBN, the overall running time of the DBN-ML was only approximately 1 h. Relative to the improvement in the classification accuracy, these time costs are completely acceptable.

Based on the results, the proposed DBN-ML model outperforms the other models. In particular, compared with the DBN-S, the model improves the OA by 0.92%. Therefore, the ML strategy produces better results than the multimodal fusion combining the DBN and MLAs. 

## 5. Conclusions

To improve the classification performance and enhance the generalization of DBN-based methods in fine LULC areas, a DBN-ML model was proposed in this study. In this DBN-ML model, the weight loss associated with the first- and second-level outputs were combined and the DBN layer used to generate the first-level output was optimized. The proposed model resulted in an OA of 95.10% and F1-score of 95.07%. Compared with other DBN- and CNN-related models, our proposed DBN-ML model proves that the multi-level output-based DBN model has a better classification effect. The classification accuracy based on the DBN-ML surpasses that reported in our previous study, which involved the DBN and CNN models. We can therefore conclude the following: (1) the ZY-3 TMS datasets can provide better feature input for a DBN-based model in open-pit mining areas and (2) the proposed two-level output-base model employed in this study is more robust, easier to interpret, and improves the fine classification accuracy. Thus, the proposed model can ensure the effective supervision of mining activities at local and regional scales. In addition, the proposed model can be used for the fine classification of other complex geo-environments. In future studies, we will focus on constructing relevant big remote sensing datasets and transferring to a learning-based model for other mining areas. 

## Figures and Tables

**Table 1:** Summary of the land cover types involved in this study and their description from [26].

First Level Type	Second Level Type	Description
Cropland	Paddy field	Adequate water supply for cultivation of aquatic crops.
	Vegetable and fruit greenhouse	High surface albedo with regular rectangular shapes.
	Dry land	On the land water resources for crops mainly from natural precipitation.
	Fallow land	No crops growing at the present stage, and for the study area, the rapeseed and wheat had just been harvested.
Forestland	Woodland	Includes timber stands, economic forests, and shelterbelts that have high chlorophyll content and are dark red in the false color image (R—NIR *, G—Red, B—Green).
	Shrub forest	Having multiple stems and shorter height, generally less than 2 m tall, and is bright red in the false color image.
	Forest under stress	Under the influence of surface mining development, around the surface-mined land, having large amounts of deposited mineral dust, has poor growth, and is grayish in the true color image (R—Red, G—Green, B—Blue).
	Nursery and orchard	Having a rectangular shape like cropland dotted by vegetation cover and exposed soil and is black in the true color image.
Water	Pond and stream	Including many fish ponds with regular rectangular shapes.
	Mine pit lake	In particular, lakes created during and after mining, normally with irregular shapes.
Road	Black road	Asphalt highways.
	White road	Cement roads.
	Gray road	Dirt roads.
Urban and rural residential land	White roof building	Urban and town areas.
	Red roof building	Rural land.
	Blue roof building	Land used for industrial parks.
Bare land	Exposed rock/soil	Exposed land with little vegetation.
Surface-mined land	Opencast stope	Having mine pit lakes and spiral roads.
	Mineral processing land	Characterized by the linear mineral processing facilities and highly reflective rubble.
	Dumping site	Located around the stope.

* NIR: near-infrared.

**Table 2:** Number and area (km^2^) of data polygons (DPs) and fractions (%) of the training, validation, and test sets. Fraction 1: fraction of pixels in the training set and DPs; Fraction 2: fraction of pixels in the validation (test) set and DPs; Fraction 3: fraction of pixels in the three sets and DPs.

Types	Number of DPs	Area of DPs (km^2^)	Training %	Validation (Test) %	Fraction 3
Paddy	43	0.14	6.41	1.60	9.61
Greenhouse	17	0.05	16.89	4.22	25.33
Green dry land	52	0.15	5.92	1.48	8.87
Fallow land	185	0.54	1.63	0.41	2.45
Woodland	57	0.54	1.63	0.41	2.44
Shrubbery	65	0.54	1.63	0.41	2.45
Coerced forest	22	0.13	6.64	1.66	9.96
Nursery	67	0.19	4.74	1.18	7.11
Pond and stream	202	0.91	0.97	0.24	1.45
Mine pit pond	33	0.05	18.40	4.60	27.60
Dark road	9	0.06	15.78	3.94	23.66
Bright road	67	0.06	14.81	3.70	22.21
Light gray road	40	0.13	6.64	1.66	9.96
Bright roof	250	0.45	1.94	0.49	2.91
Red roof	149	0.05	17.39	4.35	26.09
Blue roof	46	0.05	19.07	4.77	28.61
Bare surface	35	0.18	5.03	1.26	7.55
Open pit	44	0.13	6.56	1.64	9.84
Ore processing site	77	0.13	6.63	1.66	9.95
Dumping ground	54	0.07	13.04	3.26	19.57

**Table 3:** Classification results from the multi-level deep belief network model for various feature categories.

Category	Recall	Precision	F1- Measure
Nursery	92.40%	93.52%	92.96%
Dark road	99.40%	95.39%	97.36%
Open pit	95.80%	95.80%	95.80%
Blue roof	98.40%	98.80%	98.60%
Bright roof	84.00%	91.50%	87.59%
Red roof	94.80%	92.58%	93.68%
Greenhouse	99.00%	98.21%	98.61%
Coerced forest	97.20%	95.67%	96.43%
Fallow land	89.00%	92.90%	90.91%
Light gray road	92.00%	94.07%	93.02%
Green dry land	95.40%	93.53%	94.46%
Bare surface	95.80%	95.23%	95.51%
Woodland	94.60%	95.17%	94.88%
Dumping ground	98.40%	96.85%	97.62%
Shrubbery	90.00%	91.84%	90.91%
Paddy	99.40%	96.88%	98.12%
Mine pit pond	99.60%	99.40%	99.50%
Bright road	97.80%	91.74%	94.68%
Pond and stream	94.00%	97.92%	95.92%
Ore processing site	95.00%	94.81%	94.91%
average	95.10%	95.09%	95.07%

**Table 4:** Comparison of performance results of different models on test datasets.

Model/Evaluation Criteria	OA	Kappa	F1-Score
DBN-ML	95.10%	94.84%	95.07%
FS-SVM	91.77% ± 0.57%	91.34% ± 0.60%	91.75% ± 0.57%
DBN-S	94.23 ± 0.67%	93.93 ± 0.70%	94.22 ± 0.67%
DBN-RF	94.07 ± 0.34%	93.76 ± 0.36%	94.05 ± 0.34%
DBN-SVM	94.74 ± 0.35%	94.46 ± 0.37%	94.72 ± 0.35%
CNN	90.20% ± 1.64%	89.68% ± 1.75%	90.15% ± 1.66%
DCNN	95.02%	94.76%	95.00%

## Data Availability

Not applicable.
